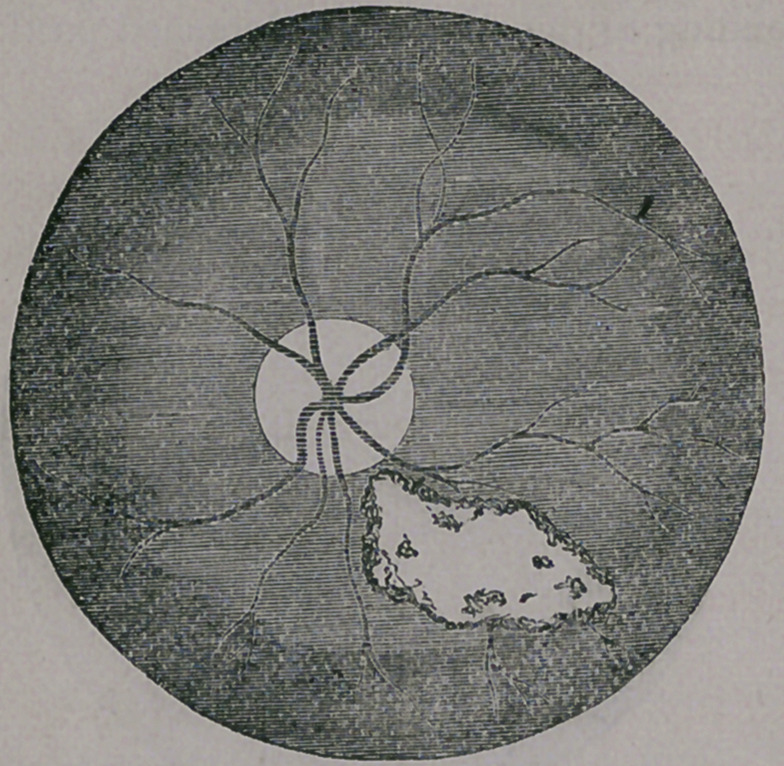# Diseases of Retina and Nerve

**Published:** 1877-01

**Authors:** 


The Bistoury.

ELMIRA, N. Y., JAN., 1877.

### DISEASES OF RETINA AND NERVE.

Many people are surprised when told that the ophthalmic surgeon can examine and plainly see the retina and nerve of the eye, and so form a correct opinion as to the nature of any malady affecting these parts. But a few years ago, when blindness ensued from diseases affecting the internal eye, the patient was said to have "amaurosis,"—a great bug-bear to both patient and physician, and was once aptly described by a celebrated physician, as being a disease in which both patient and doctor saw nothing. This somewhat comprehensive description would not answer at this day, however, when the surgeon is supplied with that wonderful instrument, the ophthalmoscope, by which he is permitted to examine the entire internal eye—not a particle of which can elude his keen observation. How this is done, and what the observer sees at such an examination, we thought would prove of interest to the readers of the BISTOURY, and we have gone to considerable expense to have the process illustrated, so that it might appear plain to the non-professional reader.

The ophthalmoscope consists simply of a concave mirror, made of finely polished steel, about two and one-half inches in diameter, and contains a small hole directly through its centre. For an examination of the eye, the patient is taken into a dark room, (the pupil of the eye having been previously dilated with a solution of atrophia) and is made to sit facing the surgeon, with a bright light burning on a level with, and immediately behind his ear. The surgeon now holds the perforated mirror before his own eye and directs the reflected rays of light coming from the lamp, upon the eye of his patient, as seen in the illustration. A double convex lens is held before the patient's eye with the left hand of the operator, so as to collect the light and cause it to pass through the pupil, and so illuminate the interior of the eye. By regulating the distance of the mirror and the lens from the eye being examined, and by causing the patient to move the eye in various directions, a complete view of its interior structure is obtained, and here is what is seen in a healthy eye:

The first object to be inspected is what is termed the *optic disk*, or, in plain English, the point at which the optic nerve and central artery and vein enter the eye. This occurs at the round, white spot, seen in the illustration. In the healthy eye, it appears round, or nearly so, and is of a yellowish white color, with a slight mixture of blue, the artery and vein entering in the centre, or, oftentimes at one side, each giving off two branches, one passing upward and the other downward, to be again severally subdivided into other branches. The retina itself, is a translucent, delicate membrane of a grayish color, but by reason of the vascular coat behind, it always appears of a rich, dark-red tint (in people with black eyes and hair) and of a more rosy hue in persons of light eyes and hair. By pressing upon the globe, the arteries are seen to pulsate so plainly that the heart's action can be readily counted. This, then, is the picture of a healthy *fundus oculi*, as the ophthalmic surgeon names it, so that it will at once appear plain to you that, should any change occur in this arrangement, that it would be at once recognized by the experienced examiner. That such changes do occur, and that, happily, we are able to account for them and often to remove them, and so prevent the most disastrous consequences to vision, we will attempt to show.

The most common change which is observed to take place in the *fundus*, is an enlargement of the vessels as well as a multiplicity of them, so that the disk, or round spot, becomes almost lost under them, as seen in our next engraving.

This condition of affairs is known as congestion of the retina, and is due to overworking the eyes, in much reading by artificial light or upon railway cars, or from sewing upon black fabrics, from watch making, use of the microscope, or from *any* cause calculated to fatigue the eye. An endeavor to read without glasses, when such auxiliaries are needed, is also a fruitful cause, and sometimes too, it is caused from certain constitutional diseases. But from whatever cause it may arise, there it is, as plain as the nose on your face, and quite as easily recognized.

Now, should the symptoms be disregarded, which usually accompany this condition of the eye—such as pains in and about the eye, sensitiveness to light, a disposition for the eyes to close when using them, lachrymation, a tired feeling always pervading them, etc., and if the patient persists in using them under this condition of affairs, the congestion is likely to bring on inflammation of the retina, accompanied with violent pain and great intolerance of light. The opthalmoscope will then show a characteristic redness of the retina, with numerous bloodvessels running all over its surface. Often, these vessels rupture, when an extravasation of blood occurs under the retina, giving rise to a large, dark colored blotch, as seen in the next illustration.

This is a very serious mishap, leaving either a black spot before the eye, or rendering it almost, or completely blind. If the patient is fortunate in securing good advice, sometimes the effused blood is absorbed, and vision partially restored; but more frequently the retina becomes detached, producing a permanent black spot before the eye, corresponding to the size of the detached portion.

Detachments of the retina occur from other causes, however—blows upon the eye, inflammation of the retina, with serous effusion, instead of blood, besides other causes not necessary to mention. But, from whatever cause it may be produced, it is regarded of the most serious consequence—should be early discovered and the means applied to absorb or remove the fluid from behind the retina, as speedily as possible. Of course, this could only be accomplished under the personal care of an experienced oculist, so that no description of the means employed becomes necessary.

The symptoms that usher in the beginning of detachment of the retina are, first, the floating gray clouds or dark spots with brilliant margins, before the eyes. These clouds or spots have wavy outlines. Again, bright flashes of light occur, followed by brilliant circles and stars. At other times, black spots and flakes seem to be floating before the eyes, which assume fantastic shapes, sometimes opening in the center, permitting the patient to see through them. They are sometimes seen for hours, then they disappear, to form some new shape for their next appearance. When the patient looks at a straight line, it seems to be wavy and broken. Sometimes these symptoms are accompanied with slight pain in the eye, but not always. To look at the external eye, no disease could possibly be detected, the pupil appearing clear, and the eye free from inflammation or unnatural appearance. But when the surgeon comes to apply his ophthalmoscope to the eye, at once the detached portion is brought to view, appearing as a white glistening blotch, upon the fundus of the eye, as seen in the next cut.

We have been particular in pointing out these difficulties of the retina, because of their danger to vision, and have minutely described the symptoms, so that they might be recognized by everyone, that incurable blindness might not follow thoughtless delay in seeking relief. Another object in thus illustrating our subject is to show the reader how readily these affections are discovered through the ophthalmoscope, and to give him some idea as to how a retina appears. Very many other diseases of the retina and nerve remain to be described, varying in appearance, symptoms and danger. But nearly or quite all of them are of a grave character, requiring early treatment to avert blindness.

## Figures and Tables

**Figure f1:**
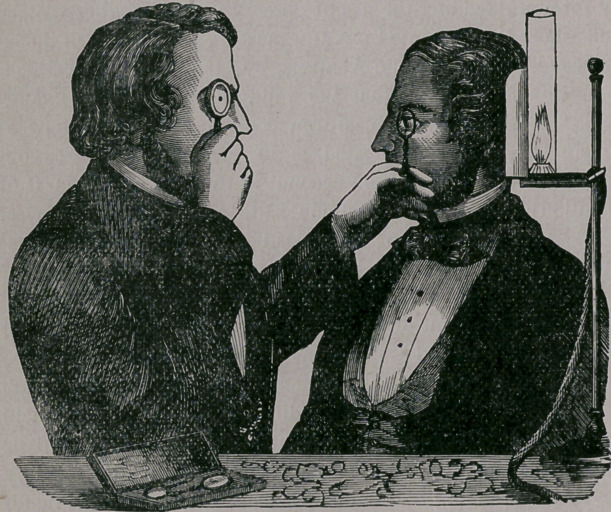


**Figure f2:**
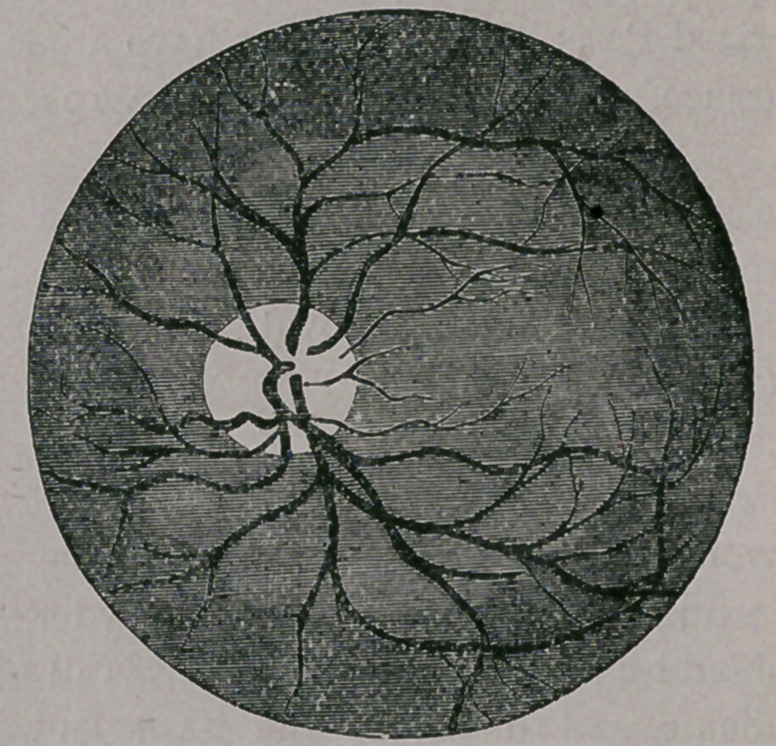


**Figure f3:**
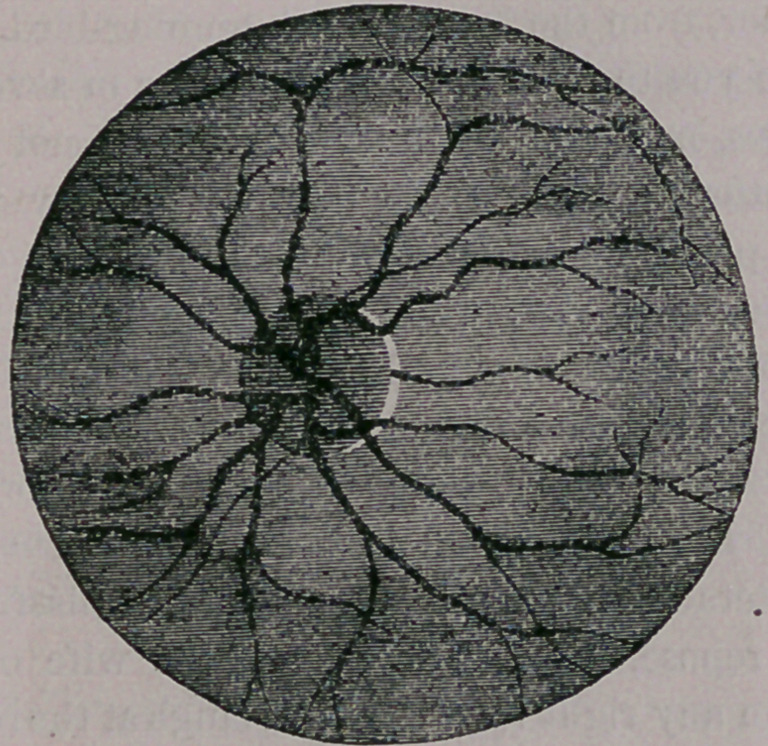


**Figure f4:**
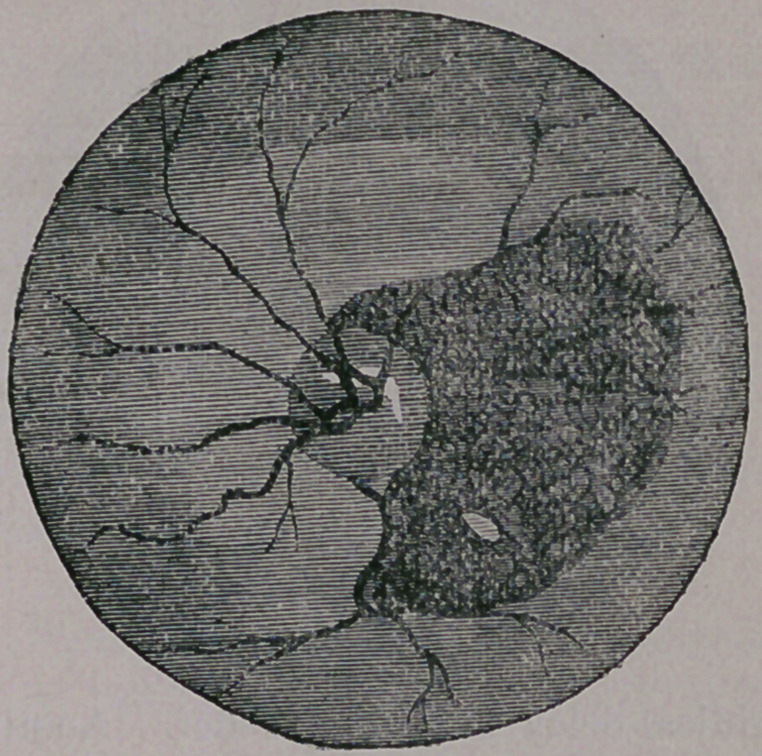


**Figure f5:**